# Precision Medicine in Epilepsy: Diagnostic Yield by Next-Generation Sequencing and Genotype–Phenotype Findings in a Romanian Cohort

**DOI:** 10.3390/ijms27198878

**Published:** 2026-10-06

**Authors:** Lăcrămioara Ionela Butnariu, Ecaterina Grigore, Thomas Gabriel Schreiner, Aurora Băgiag, Setalia Popa, Ioana Grigore

**Affiliations:** 1 Grigore T. Popa University of Medicine and Pharmacy, 700115 Iasi, Romania; egrigore2002@gmail.com (E.G.); thomas.schreiner@umfiasi.ro (T.G.S.); setalia.popa@umfiasi.ro (S.P.); 2 Regional Center for Medical Genetics Iasi, Saint Mary’s Emergency Children Hospital, 700309 Iasi, Romania; 3 Iuliu Haţieganu University of Medicine and Pharmacy, 400012 Cluj-Napoca, Romania; aurora.bagiag@umfcluj.ro; 4 Neurology, Saint Mary’s Emergency Children Hospital, 700309 Iasi, Romania; ioanag74@yahoo.com

**Keywords:** genetic epilepsy, next-generation sequencing, genotype–phenotype findings, precision medicine, developmental and epileptic encephalopathy, variants of uncertain significance, ion channel genes, copy number variations, whole-exome sequencing, Romanian cohort

## Abstract

Epilepsy ranks among the most prevalent neurological disorders globally, with a substantial proportion of cases, particularly in pediatric populations, attributed to genetic etiologies. This study provides the first molecular characterization of suspected genetic epilepsy in a pediatric cohort from the Moldova region of Romania. Following the exclusion of 124 patients with secondary acquired or structural causes from an initial cohort of 159, we enrolled 35 individuals with a high suspicion of primary genetic epilepsy, who underwent targeted gene panel testing or whole-exome sequencing (WES). Pathogenic (P) or likely pathogenic (LP) variants were identified in 13 patients (37.1%); variants of uncertain significance (VUSs) were identified in another 13 patients (37.1%), comprising 15 distinct VUSs; and negative results were observed in the remaining 9 patients (25.7%). Genes regulating ion channels and synaptic signaling, notably *SCN1A*, *KCNB1*, and *GNAO1*, were predominantly implicated in early-onset, drug-resistant epileptic encephalopathies, whereas structural copy number variations (CNVs) underpinned syndromic phenotypes. We identified one novel pathogenic variant in *SCN1A*, thereby expanding the known mutational spectrum. Furthermore, in silico predictions and de novo occurrence patterns supported the pathogenicity of several VUSs, underscoring the critical value of familial segregation analyses. Despite the notable diagnostic yield, the high VUS rate underscores the ongoing need for refined variant interpretation criteria and the integration of functional validation data to improve classification accuracy. Collectively, our findings support the clinical utility of next-generation sequencing (NGS) in informing therapeutic decisions, enabling genetic counseling, and improving prognostic accuracy. These results suggest that NGS should be considered early in the diagnostic workup of unexplained or drug-resistant epilepsy, particularly in patients with clinical features suggestive of a genetic etiology.

## 1. Introduction

**Figure 1:**
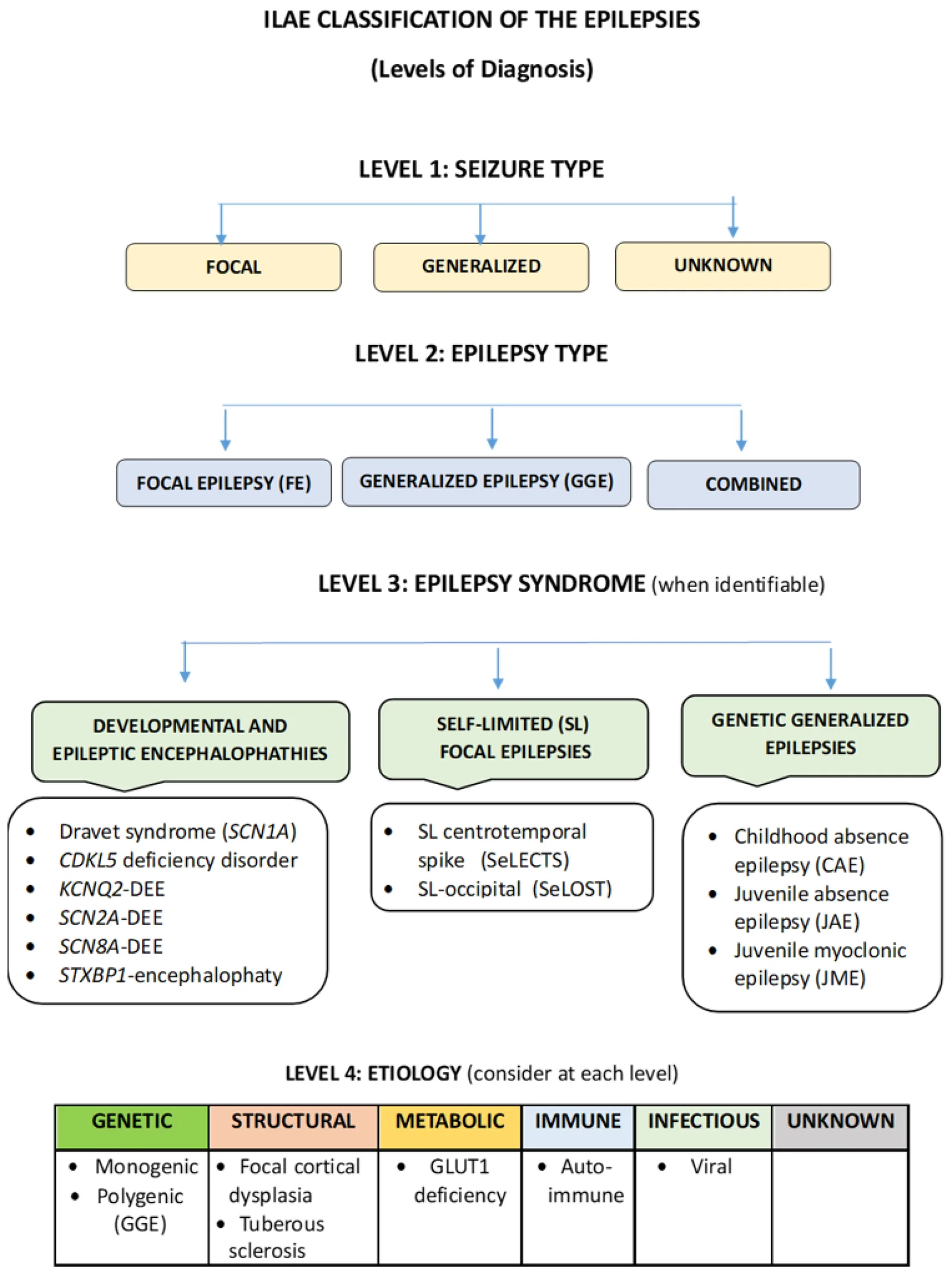
Four-level diagnostic framework for epilepsy according to the International League Against Epilepsy (ILAE) [10,11,12].The classification proceeds from seizure type (Level 1) to epilepsy type (Level 2) and, when possible, to epilepsy syndrome (Level 3). Etiology (Level 4) must be considered at each diagnostic level. Selected genetic etiologies with their associated genes and syndromes are provided as illustrative examples. GGE: genetic generalized epilepsy; DEE: developmental and epileptic encephalopathy; GTCS: generalized tonic–clonic seizures alone; SeLECTS: self-limited centro-temporal spikes; SeLOST: self-limited occipital spikes. Adapted from ILAE 2017/2022/2025 classifications with integration of genetic data.

Epilepsy affects approximately 50 million individuals worldwide and imposes a substantial clinical, social, and economic burden, with a particular predilection for childhood, where it affects 0.5% to 1% of children [1,2,3,4,5]. Although its etiological landscape is remarkably heterogeneous, its genetic architecture has increasingly become the cornerstone of modern neuroscientific research [6,7]. The proportion of epilepsy cases attributable to genetic etiologies has grown substantially with advances in molecular diagnostics. Current estimates indicate that genetic factors play a primary or contributing role in a significant fraction of epilepsies, particularly in early-onset and syndromic presentations [8]. Genetic factors are now recognized as the cause in an estimated 30% of all epilepsies [9]. The International League Against Epilepsy (ILAE) classification system recognizes genetic epilepsies as a distinct etiological category, acknowledging that pathogenic variants can directly cause or predispose one to seizure disorders [10,11,12] (Figure 1).

From a syndromic classification perspective, the ILAE has established a hierarchical framework that integrates genetic findings with clinical phenotype, subdividing childhood-onset syndromes into self-limited focal epilepsies, genetic generalized epilepsies (GGEs), and developmental and/or epileptic encephalopathies (DEEs) (Figure 1) [10,11,12].

Monogenic etiologies explain about 20–40% of recruited cases with early-onset epilepsies in different studies [13,14,15], while among adults with epilepsy and co-occurring neurodevelopmental disorders, the diagnostic yield ranges from 23% to 50% [16].

The genetic architecture of monogenic epilepsies is characterized by significant genetic heterogeneity, predominantly featuring mutations in genes regulating neuronal excitability and synaptic integrity. *SCN1A*, encoding the Nav1.1 voltage-gated sodium channel, remains the most frequently implicated gene across both pediatric and adult populations [16,17]. Beyond *SCN1A*, commonly identified variants are found in other ion channel genes, including *KCNQ2*, *KCNT1*, *SCN2A*, and *SCN8A* [14,18], as well as non-channel genes such as *STXBP1*, *PCDH19*, *SYNGAP1*, *CHD2*, and *SLC2A1* [10,19]. Identifying these variants is increasingly vital for precision medicine, as it enables gene-driven therapeutic strategies and informs long-term clinical management [14,16].

While monogenic mutations cause most severe, early-onset epilepsies [13,14], common epilepsies, including genetic generalized epilepsy (GGE) and non-acquired focal epilepsy (NAFE), have a complex, predominantly polygenic or oligogenic etiology [20,21,22]. This architecture involves the cumulative effect of numerous common genetic variants (polygenic risk), each with a small individual risk contribution, alongside rare variants [22,23,24].

Genome-wide association studies (GWAS) have identified 26 significant loci for epilepsy (including 29 likely causal genes), 19 of which are specific to GGEs, with SNP-based heritability analyses showing that common variants explain between 39.6% and 90% of the genetic risk for GGEs [24]. Several genes first identified by the common variant GWAS (e.g., *SCN1A*, *SCN8A*, *DEPDC5*) are also established monogenic epilepsy genes, demonstrating biological convergence between common polygenic and rare monogenic epilepsies [20,21,25,26].

Polygenic risk scores (PRS) are emerging as a promising biomarker for epilepsy, particularly for genetic generalized epilepsy (GGE), by aggregating the minor effects of thousands of common DNA variants [27]; while they may aid risk stratification after a first unprovoked seizure, they are not yet recommended for routine clinical diagnostics due to limitations in predictive accuracy and ancestral diversity [21,27]. 

A recent meta-analysis showed that individuals in the top 0.5% of the PRS distribution have an odds ratio of 4.62 (95% CI 3.45–6.20) for developing GGE, compared with 1.69 (95% CI 1.27–2.24) for focal epilepsy [28].

Beyond risk prediction, higher epilepsy PRS correlates with more severe phenotypes in relatives sharing the same pathogenic variant, supporting a model where rare and common variants interact to shape disease expression [23]. Current limitations include restricted validation to European-ancestry populations, modest predictive accuracy (c-index ~0.60) [29], and significant study heterogeneity [28]. Nevertheless, integrating PRS with clinical variables holds promise for personalized risk prediction and targeted treatment guidance [23].

Establishing genotype–phenotype correlations remains a challenge in epilepsy management and for future research. The phenomenon of genetic heterogeneity explains both the emergence of distinct phenotypes caused by different allelic variants of the same gene and the convergence of similar phenotypes resulting from mutations in different genes [13,14,30]. For instance, loss-of-function (LOF) mutations (nonsense or frameshift) in the *SCN1A* gene cause Dravet syndrome, a severe infantile-onset epileptic encephalopathy, whereas missense variants of the same gene can manifest as genetic epilepsy with febrile seizures plus (GEFS+), which is a milder phenotype with a favorable prognosis [14,17,30].

Next-generation sequencing (NGS), including multigene panels and whole-exome sequencing (WES), has rapidly moved from a research tool to a standard-of-care investigation for people with unexplained, early-onset, or drug-resistant epilepsy [31,32]. The evidence to date demonstrates that molecular diagnosis identifies the causative genetic variant in approximately 25–40% of cases [31]. This has a direct impact on clinical management, improves patient outcomes, and ends the ‘diagnostic odyssey’ for many families [31,32]. Furthermore, gene discovery provides the basis for neurobiological insights and the development of targeted therapies [33].

Currently, genetic testing by NGS (especially WES) is recommended as a first-line method for children with epilepsy of an unknown cause, having a major impact on clinical management [34,35].

Beyond diagnosis, identifying specific genetic etiologies carries direct therapeutic implications, marking the advent of precision medicine in epilepsy.

Targeted strategies include the ketogenic diet for GLUT1 deficiency syndrome (*SLC2A1*), sodium channel blockers for gain-of-function (GOF) mutations in *KCNQ2*, *SCN2A*, and *SCN8A*, and mTOR inhibitors for mTORopathies such as tuberous sclerosis complex (*TSC1*/*TSC2*) [36]. As the repertoire of genetically guided treatments expands, early and comprehensive genetic testing becomes a prerequisite for optimized, mechanism-based management [32,37]. Gene therapy holds promise for focal, generalized, and genetic epilepsies, but its success will depend on smarter trial designs, better biomarkers, and refined technologies such as improved viral vectors, tighter gene expression controls, and RNA-based tools that directly target seizure causes [38].

To date, no comprehensive molecular characterization of genetic epilepsies has been performed in pediatric cohorts from the Moldova region of Romania.

To address this knowledge gap and to provide the first molecular data on genetic epilepsies in this understudied region, this study aims to characterize the genetic landscape of a Romanian cohort of patients with suspected genetic epilepsy from the Moldova region utilizing next-generation sequencing (NGS). The primary objectives are to evaluate the diagnostic yield, describe genotype–phenotype findings, and demonstrate the clinical utility of molecular testing in guiding therapeutic decisions, improving prognostic accuracy, and enabling precise genetic counseling. By elucidating these relationships, this research seeks to enhance risk stratification for severe clinical phenotypes and support personalized management strategies.

## 2. Results

### 2.1. Cohort Characteristics and Genetic Testing Yield

From an initial cohort of 159 patients diagnosed with epilepsy between January 2024 and June 2026 at the Neurology Clinic and Regional Genetic Center of St. Mary Emergency Children Hospital in Iași, 124 were excluded due to clear secondary acquired or structural etiologies. Of the 35 patients selected for genetic testing based on the predefined criteria (see Materials and Methods), the most frequent indications were an early age at onset (*n* = 27, 77.1%), drug-resistant epilepsy (*n* = 22, 62.9%), neurodevelopmental comorbidities (*n* = 19, 54.3%), dysmorphic features or congenital anomalies (*n* = 14, 40.0%), and a positive family history (*n* = 4, 11.4%). Patients could meet more than one inclusion criterion. Of the 35 patients, 17 underwent whole-exome sequencing (WES), and 18 underwent targeted epilepsy gene panel testing. WES identified pathogenic or likely pathogenic (P/LP) variants in 7/17 patients (41.2%) and variants of uncertain significance (VUSs) in 6/17 patients (35.3%), with negative results in 4/17 patients (23.5%). Targeted gene panel testing identified P/LP variants in 6/18 patients (33.3%) and VUS in 7/18 patients (38.9%), with negative results in 5/18 patients (27.8%). Overall, the diagnostic yield for P/LP variants was 37.1% (13/35), with an additional 37.1% (13/35) of patients carrying a VUS. The distribution of P/LP and VUSs by testing method is summarized in Table S1 (Supplementary Material).

NGS-based evaluation allowed stratification into three categories: a molecularly confirmed (P/LP) cohort (*n* = 13, 37.1%) (Table 1); a VUS cohort (*n* = 13, 37.1%) (Table 2); and a negative cohort (*n* = 9, 25.7%). The 13 patients with a VUS were excluded from the primary genotype–phenotype characterization due to the inconclusive nature of their molecular findings, but their molecular data are presented separately in Table 2)) to highlight potential phenotypic overlaps and to underscore the need for future variant reclassification. The nine patients with negative results (in whom molecular testing revealed no reportable variants) were not included in further analyses. A descriptive comparison showed that these patients had a lower frequency of dysmorphic features and congenital anomalies than the genetically positive groups; however, given the small sample size and the absence of formal statistical testing, no conclusions can be drawn regarding the relationship between systemic manifestations and the likelihood of identifying a genetic etiology.

### 2.2. Mutational Profile and Classification of P/LP Variants

Detailed analysis of the 13 patients with molecularly confirmed diagnoses (Table 1) revealed a predominance of point mutations (SNVs/indels, *n* = 10) compared to structural variants (CNVs, *n* = 3).

Among the SNV/indel variants, we identified four missense mutations: P02 (M.P.): *GNAO1* c.607G>A p.(Gly203Arg); P03 (C.I.): *GNAO1* c.143C>T p.(Thr48Ile); P10 (C.M.): *CACNA1A* c.4046G>A p.(Arg1349Gln); and P11 (A.M.): *AFF3* c.697G>A p.(Ala233Thr). Four nonsense mutations (stop_gained) were found in P01 (M.I.): *KCNB1* c.1107G>A p.(Trp369*); P05 (P.C.): *SCN1A* c.1837C>T p.(Arg613Ter); P06 (B.A.): *SCN1A* c.2870G>A p.(Trp957*); and P13 (T.L.): *CSDE1* c.1087C>T p.(Arg363*). One frameshift mutation was identified in P04 (B.T.), *GPHN* c.1206del p.(Ala403Hisfs3*), and one splice-site mutation in P12 (B.M.), *GRIN2A* c.1007+1G>A, with a predicted effect on the splice donor (p.?).

Of the 13 P/LP variants, 12 are known (previously reported in ClinVar or the literature), while one nonsene variant is novel—*SCN1A* c.2870G>A, p.(Trp957*)—in patient P06 (Table 1).

Structural variants (CNVs) were detected in three patients. These included a 15q11.2-q13.1 microdeletion involving the *GABRB3* and *UBE3A* genes (P07, B.D.), a 17q21.31 microdeletion involving exons 3–15 of *KANSL1* (P08, N.V.), and a complex chromosomal rearrangement consisting of an 8p inversion/duplication/deletion (P09, G.I.). For these structural variants, protein-level consequences are not applicable (Table 1).

All SNV/indel P/LP variants were identified in a heterozygous state, consistent with an autosomal dominant inheritance pattern for the involved genes. The structural variants had distinct inheritance mechanisms: the 15q11.2-q13.1 deletion involved an imprinted region, with maternal origin confirmed by methylation analysis, whereas the 8p rearrangement was a complex chromosomal rearrangement consistent with inverted duplication/deletion of 8p, based on the characteristic pattern of terminal deletion and inverted duplication.

A family history of epilepsy was negative in 12 out of the 13 patients (92.3%), with patient P01 (M.I.), *KCNB1* c.1107G>A p.(Trp369*), being the sole exception (Table 1). However, parental testing was not performed in most cases; therefore, de novo status could not be confirmed and should be considered presumed rather than established.

### 2.3. In Silico Predictive Analysis of Variants of Uncertain Significance (VUSs)

For the 13 patients with a VUS classified according to ACMG/AMP guidelines (Table 2), comprehensive in silico analysis was performed to assess potential functional impact. CADD scores exceeded 20 in 8 of the 15 variants, and REVEL scores were above 0.75 in 7 of the analyzable missense variants. SIFT predicted most variants as deleterious, except for those in P20 (*HECW2* c.4492C>A, p.(Gln1498Lys), P21 (*RORA* c.615C>G, p.(Asp205Glu), and P22 (*ANKRD11* c.6209A>G, p.(Lys2070Arg), which were classified as tolerated. Allelic frequencies in gnomAD were extremely low or absent (range 0.000000–0.0000428), supporting their rarity and the need for further functional investigation.

The VUS cohort displayed greater zygosity diversity compared to the P/LP group. Beyond heterozygosity, we identified two homozygous variants (*TBC1D24* c.448G>A, p.(Glu150Lys) in P18; *TBCD* c.3250G>T, p.(Asp1084Tyr) in P19 and two X-linked hemizygous variants in male patients: *CNKSR2* c.1709G>A, p.(Arg570His) in P16, and *ARX* c.499G>A, p.(Val167Met) in P24. Segregation analysis confirmed de novo origins for four variants: two previously reported *LGI1* c.1597C>T, p.(Arg533Cys) in P15 and *ANKRD11* c.6209A>G, p.(Lys2070Arg) in P22; and two novel variants, *KCNQ2* c.317G>A, p.(Cys106Tyr) in P14 and *RORA* c.615C>G, p.(Asp205Glu) in P21. For the remaining variants, inheritance could not be established due to unavailable parental samples.

Overall, 15 distinct VUSs were identified across 13 patients, spanning 14 genes and highlighting substantial genetic heterogeneity with a predominance of heterozygosity (Table 2). Four variants were previously reported: *LGI1* c.1597C>T, p.(Arg533Cys) in P15; *CACNA1E* c.3275C>T, p.(Thr1092Met) in P17; *TBC1D24* c.448G>A, p.(Glu150Lys) in P18; and *CACNA1A* c.1954G>A, p.(Asp652Asn) in P25. The remaining 11 variants in nine patients were novel: *KCNQ2* c.317G>A, p.(Cys106Tyr) in P14; *CNKSR2* c.1709G>A, p.(Arg570His) in P16; *TBCD* c.3250G>T, p.(Asp1084Tyr) in P19; *HECW2* c.4492C>A, p.(Gln1498Lys) in P20; *RORA* c.615C>G, p.(Asp205Glu) in P21; *ANKRD11* c.6209A>G, p.(Lys2070Arg) in P22; *TRPM3* c.1262A>G, p.(Tyr421Cys) in P23; *POLR2A* c.4720T>C, p.(Ser1574Pro) in P23; *ARX* c.499G>A, p.(Val167Met) in P24; *CHRNB2* c.1082T>C, p.(Leu361Pro) in P26; and *CNKSR2* c.1154A>T, p.(His385Leu) in P26. Zygosity analysis revealed two homozygous variants (*TBC1D24* in P18; *TBCD* in P19) and two X-linked hemizygous variants (*CNKSR2* in P16; *ARX* in P24). Segregation analysis confirmed de novo origins for four variants: two previously reported (*LGI1* in P15; *ANKRD11* in P22) and two novel (*KCNQ2* in P14; *RORA* in P21). For the remaining variants, inheritance could not be established due to unavailable parental samples.

Of particular note, two patients (P23 and P26) harbored two distinct VUS each: P23 had two novel variants (*TRPM3* and *POLR2A*), while P26 had one previously reported variant (*CHRNB2*) and one novel variant (*CNKSR2)* (Table 2). These patients carry multiple VUSs, and their clinical significance remains to be determined. The in silico predictions, combined with low population allele frequencies, support the need for further investigation to clarify their potential clinical significance.

Detailed clinical information, including age at seizure onset, epilepsy type, EEG characteristics, response to antiseizure therapy, and other associated systemic manifestations, is provided in Table 3 for the P/LP cohort and in Table 4 for the VUS cohort.

### 2.4. Descriptive Comparative Analysis of Clinical Characteristics

A comparative evaluation between the P/LP cohort and the VUS cohort revealed significant phenotypic overlaps, alongside notable differences between the two groups (Table 5).

#### 2.4.1. EEG Findings, Seizure Types, and Response to Antiseizure Medication

In both cohorts, EEG abnormalities were severe, dominated by generalized or focal spike–slow wave complexes, with a predilection for centro-temporal or fronto-temporal regions (Table 3 and Table 4).

Regarding responses to antiseizure medication (ASM), in the P/LP cohort, good seizure control (GR) was observed, even in patients with severe disease courses, such as P02 (M.P.) *GNAO1* c.607G>A, p.(Gly203Arg); P03 (C.I.) *GNAO1* c.143C>T, p.(Thr48Ile); and P07 (B.D.) Angelman syndrome, 15q11.2-q13.1 deletion. In contrast, therapeutic resistance (UR) was marked in patient P01 (M.I.) *KCNB1* c.1107G>A, p.(Trp369*); and P08 (N.V.) Koolen–de Vries syndrome (17q21.31 deletion).

Patients in the VUS cohort showed a higher rate of unsatisfactory treatment response (UR) (30.8%, *n* = 4/13), failing to achieve adequate seizure control despite receiving multiple antiseizure medications, especially in those carrying variants affecting ion channels: P14 (S.V.): *KCNQ2* c.317G>A, p.(Cys106Tyr); P25 (L.S.): *CACNA1A* c.1954G>A, p.(Asp652Asn); P26 (B.E.): *CHRNB2* c.1082T>C, p.(Leu361Pro); or synaptic secretion proteins: P15 (H.S.): *LGI1* c.1597C>T. p.(Arg533Cys).

#### 2.4.2. Associated Systemic Manifestations and Comorbidities

Intellectual disability (ID) and autism spectrum disorders (ASD) represented major comorbidities in both groups, being observed in association with genes involved in synaptogenesis and neuronal excitability (*SCN1A*, *ARX*, *CNKSR2*, *RORA*) (Table 3 and Table 4).

In contrast, non-neurological and dysmorphic features were both more frequent and more severe in the P/LP cohort. Severe proportionate dwarfism (ranging from −2.5 to −3.58 SD) and complex craniofacial dysmorphism were prominent findings in this group, particularly in patients P01 (M.I.): *KCNB1* c.1107G>A, p.(Trp369*); P03 (C.I.): *GNAO1* c.143C>T, p.(Thr48Ile); P09 (G.I.): 8p inverted duplication/deletion syndrome; and P13 (T.L.): *CSDE1* c.1087C>T, p.(Arg363*).

Organ malformations, including coarctation of the aorta, atrial septal defect, patent ductus arteriosus, and severe skeletal anomalies (polydactyly, metatarsal agenesis) were documented exclusively in the cohort with definite pathogenic variants, particularly in P11 (A.M.): *AFF3* c.697G>A p.(Ala233Thr) with KINS syndrome, underscoring the multisystemic nature of these genetic entities compared to the predominantly neurodevelopmental clinical picture observed in the VUS cohort.

### 2.5. Patients with Negative Genetic Testing Results

The nine patients in whom genetic testing did not identify any pathogenic, likely pathogenic, or VUS variants were not included in the subsequent genotype–phenotype correlation analyses. Nevertheless, they remain eligible for future re-evaluations through next-generation sequencing methods, such as whole-genome sequencing (WGS), or through periodic updates of variant databases and reclassification.

## 3. Discussion

This study provides a comprehensive molecular and clinical characterization of a Romanian cohort of patients with suspected genetic epilepsy, offering insights into the diagnostic yield of next-generation sequencing (NGS), the spectrum of pathogenic variants and variants of uncertain significance (VUSs), and the genotype–phenotype correlations that underpin the heterogeneous clinical presentations observed in this population.

### 3.1. Diagnostic Yield and Clinical Utility of Genetic Testing

In our cohort, extended genetic testing identified pathogenic or likely pathogenic (P/LP) variants in 13 out of 35 patients (37.1%), selected based on a high clinical suspicion of a genetic etiology. This yield is consistent with previously reported rates (25–40%) in early-onset or drug-resistant epilepsies [40,41] and aligns with the upper end of this range, underscoring the importance of careful patient selection [42]. The remaining 13 patients (37.1%) carried a VUS, while 9 (25.7%) had negative results, highlighting the ongoing challenges in variant interpretation and the need for periodic re-evaluation as databases evolve.

A retrospective study by Chourasia et al. [43], analyzing 1025 pediatric epilepsy patients, demonstrated that 8% experienced at least one genetic variant reclassification, with 19% of the re-evaluated VUSs being upgraded to pathogenic or likely pathogenic status [43]. These findings underscore that an initial genetic report should not be treated as a static endpoint, supporting routine periodic re-evaluation in clinical practice, as recommended by the ILAE Genetics Commission [42].

The identification of a molecular diagnosis had direct implications for clinical management, enabling more accurate prognostication, informing reproductive counseling, and, in some cases, guiding therapeutic decisions. For example, the diagnosis of Dravet syndrome in patients P05 and P06 (*SCN1A*) prompted recommendations to avoid sodium channel blockers, which can exacerbate seizures in this condition [44,45]. Similarly, the identification of *KCNB1*-related DEE26 in patient P01 reinforced the need for early, aggressive seizure management and surveillance for neurodevelopmental comorbidities [46]. These observations support the emerging consensus that genetic testing should be considered early in the diagnostic workup of children with unexplained, early-onset, or pharmacoresistant epilepsy [47].

### 3.2. Mutational Spectrum and Genotype–Phenotype Findings

The mutational landscape of our P/LP cohort reflects the well-established genetic heterogeneity of monogenic epilepsies [13,14]. Ion channel genes predominated, with *SCN1A* (*n* = 2), *GNAO1* (*n* = 2), *KCNB1* (*n* = 1), and *CACNA1A* (*n* = 1) accounting for nearly half of the identified variants (Table 1).

This distribution aligns with data from a large-scale genomic cohort and comprehensive literature reviews [46,48,49], which collectively highlight that the etiology of early-onset epileptic encephalopathies is driven both by channelopathies affecting voltage-gated sodium (*SCN1A*), potassium *(KCNB1)*, and calcium channels (*CACNA1A*) [46,48,49,50,51], and by dysfunctions in G-protein-mediated intracellular signaling pathways (*GNAO1)* [50,52,53].

#### 3.2.1. *GNAO1* c.607G>A (p.Gly203Arg) and c.143C>T (p.Thr48Ile) Variants

Pathogenic variants in *GNAO1* are associated with severe neurodevelopmental phenotypes, including early-onset epileptic encephalopathy, hyperkinetic movement disorders, and profound developmental delay [53]. The c.607G>A p.(Gly203Arg) variant is a well-characterized mutational hotspot linked to refractory epilepsy and chorea, with seizure onset within the first months of life and profound global motor delay [54]. Multiple case series have documented treatment-resistant dystonic storms in affected individuals, including four patients reported by Arya et al. [54] and two additional unrelated female patients described by Schorling et al. [55].

At the molecular level, the c.607G>A substitution lies within the highly conserved switch II region of the Gαo protein, impairing GTP binding and downstream effector activation [55]. Functional studies have shown that the Gαo-G203R mutant loses its ability to enhance cAMP synthesis and exerts a dominant-negative effect on neuronal activity and GABAB-dependent synaptic release [56,57,58]. This variant is classified as pathogenic in ClinVar (Variation ID: 66115) [59]. Our patient presented a comparable phenotype, albeit without non-epileptic spasms, reinforcing the phenotypic variability that can occur even among individuals harboring the same mutation [50,54].

The c.143C>T p.(Thr48Ile) variant, though less frequently reported, is a well-established pathogenic mutation linked to DEE17 (OMIM 615473) [39] with burst-suppression, a pattern characteristic of Ohtahara syndrome [52,55]. Initially identified in a patient with severe epileptic encephalopathy [55], it has since been confirmed to occupy the severe end of the phenotypic spectrum, including early-onset refractory epilepsy and, in some cases, predominantly movement disorders [52,55]. This variant is classified as pathogenic in ClinVar (Variation ID: 435341) [59], underscoring its deleterious impact on Gαo protein function and neuronal signaling [55]. Our patient exhibited the severe encephalopathic phenotype with burst-suppression, confirming prior observations by Schorling et al. [55].

The broader phenotypic spectrum of *GNAO1*-related disorders ranges from severe forms with intractable epilepsy and prominent movement abnormalities (including Ohtahara syndrome and choreoathetosis) to predominantly dystonic phenotypes that may occur in the absence of seizures [49,53]. Identification of these variants has important implications for therapeutic decision-making, including consideration of deep brain stimulation for refractory dystonia, and for genetic counseling of affected families [53,54].

#### 3.2.2. *SCN1A*-Related Dravet Syndrome: Clinical Spectrum and Novel Variants

*SCN1A* mutations, the most common monogenic cause of Dravet syndrome [17], were identified in two patients (P05 and P06), both presenting with classic features, including febrile seizures, myoclonic seizures, and moderate intellectual disability with autism spectrum traits. Patient P05 carried the known pathogenic nonsense variant c.1837C>T, p.(Arg613Ter), registered in ClinVar (Variation ID: 93635) [59] and previously reported in the literature [17,30].

Patient P06 harbored a novel nonsense variant c.2870G>A, p.(Trp957*), which expands the mutational spectrum of *SCN1A* and underscores the utility of whole-exome sequencing (WES) in detecting previously unreported loss-of-function alleles. Both nonsense variants are predicted to result in loss-of-function through nonsense-mediated mRNA decay, leading to haploinsufficiency of the Nav1.1 sodium channel, consistent with the severe Dravet syndrome phenotype [17,45].

The frequency of *SCN1A* mutations in our cohort (2/13, 15.4%) is consistent with international studies. In a Chinese cohort of 283 patients with unexplained epilepsy, Hu et al. [31] identified *SCN1A* as the predominant pathogenic gene, accounting for 14 of 81 (17.3%) variants detected [31]. A Polish study by Chałupczyńska et al. [60] reported *SCN1A* as one of the most frequent contributors, accounting for 12.6% (11/87) of positive diagnoses in a pediatric cohort [60].

Collectively, these data confirm *SCN1A* as the most frequently implicated gene in monogenic epilepsies, with the highest mutation frequencies consistently reported in cohorts enriched for Dravet syndrome and early-onset developmental and epileptic encephalopathies (DEEs) [14,17,61]. Our findings appear consistent with this established pattern, though the small size of our cohort (*n* = 13 with P/LP variants) limits the generalizability of our observations. The identification of a novel *SCN1A* nonsense variant highlights the potential utility of comprehensive sequencing approaches in uncovering population-specific allelic diversity, yet larger, independent studies are required to validate this finding and to delineate the full mutational spectrum of *SCN1A* in the Romanian population. Until such validation is available, our results should be interpreted as preliminary and hypothesis-generating rather than definitive.

#### 3.2.3. *KCNB1* Encephalopathy: The c.1107G>A, p.(Trp369*) Nonsense Variant

The heterozygous nonsense variant c.1107G>A (p.Trp369*) in the final exon of *KCNB1* confirms the diagnosis of DEE26 in patient P01. This variant is classified as pathogenic in ClinVar (Variation ID: 1427157) [59] and represents a private nonsense allele, highlighting the allelic heterogeneity of *KCNB1*-related disorders [62,63]. The terminal location suggests that the mutant mRNA transcript escapes nonsense-mediated decay (NMD), potentially producing a truncated Kv2.1 channel protein lacking 490 amino acids with a dominant-negative effect [62]. This contrasts with truncating variants in earlier exons, which are more likely to be eliminated by NMD and result in haploinsufficiency [62]. The clinical presentation of patient P01 (severe intellectual disability, drug-resistant focal epilepsy, autism spectrum disorder, and absent speech) is consistent with *KCNB1* encephalopathy [62]. In the cohort of de Kovel et al. [63], developmental delay and intellectual disability were universally reported, alongside epilepsy in 84% of cases and behavioral issues in 82% [63]. The review by Bar et al. [62] reaffirms the broad neurodevelopmental spectrum of this disease, emphasizing language difficulties and neuropsychiatric impairment [62]. Truncating variants in the C-terminal domain are generally associated with less severe epilepsy but substantial neuropsychiatric and behavioral deficits [62]. Although patient P01’s seizures demonstrated drug resistance, the overall clinical picture aligns with this tendency. The molecular confirmation of this variant, presumed to be of de novo origin, enables accurate genetic counseling and guides anticipatory management of associated comorbidities [62,63]. While targeted therapies for Kv2.1 dysfunction are unavailable, optimization of antiseizure medication and early therapeutic intervention remain the cornerstones of patient management. Further functional studies, such as those suggested by Xiong et al. [64], are required to elucidate the pathogenic mechanism of this variant and to refine prognostic models [64].

#### 3.2.4. Structural Variants: Microdeletion Syndromes in Epilepsy

Structural variants (CNVs) were identified in three patients with well-characterized microdeletion syndromes: Angelman syndrome (P07), Koolen–de Vries syndrome (P08), and 8p inverted duplication/deletion syndrome (P09), each combining epilepsy and intellectual disability with dysmorphic features or congenital anomalies.

Patient P07 harbored a 5.5 Mb deletion in 15q11.2-q13.1 encompassing *GABRB3* and *UBE3A*. The clinical presentation—generalized tonic–clonic seizures (onset at 2 years 3 months), motor delay, and ataxia—aligns with Angelman syndrome [65]. Chromosomal deletions typically yield more severe phenotypes than single-gene mutations due to co-deletion of adjacent GABA receptor genes *(GABRB3*, *GABRA5*, and *GABRG3*) [66,67], consistent with seizures affecting 80–90% of individuals with Angelman syndrome (onset 12–18 months) [68]. The epileptic phenotype is further exacerbated in chromosomal deletions, which confer increased seizure susceptibility and treatment resistance [66,67].

Patient P08 presented with a 310.5 kb deletion in 17q21.31, disrupting exons 3–15 of *KANSL1*, confirming Koolen–de Vries syndrome (KVS) and accounting for early-onset seizures (at 9 months), severe intellectual disability, cryptorchidism, and atrial septal defect [69,70]. While seizures occur in approximately one-third (33%) of affected individuals [70], *KANSL1* haploinsufficiency disrupts chromatin-remodeling pathways, leading to multisystemic neurodevelopmental phenotypes [69,70].

Patient P09 carried a complex rearrangement on chromosome 8p (deletion 8p23.3p23.1 and duplication 8p23.1p11.21), characteristic of 8p inverted duplication/deletion syndrome [71]. The severe intellectual disability, spastic paraparesis, extrapyramidal signs, complex cardiac malformation, and partial agenesis of the corpus callosum represent a textbook manifestation of this rearrangement [71,72]. The literature confirms developmental delay is near-ubiquitous (97%), while corpus callosum defects occur in 63% of cases [72].

Taken together, these cases underscore the clinical utility of WES as a dual-purpose tool that captures both single-nucleotide and structural variants [32]. Our identification of rearrangements, ranging from 5.5 Mb to 310.5 kb, demonstrates that WES can streamline the diagnostic workup of epilepsy [14], particularly when seizures coexist with syndromic traits, developmental delay, or congenital anomalies [34]. The diverse multisystemic manifestations reinforce that CNVs affecting dosage-sensitive genes exert pleiotropic effects beyond the central nervous system, where an early WES-based diagnosis can accelerate targeted multidisciplinary management [14,32].

#### 3.2.5. *CACNA1A*-Related DEE42: The c.4046G>A, p.(Arg1349Gln) Missense Variant

Patient P10 harbors the heterozygous missense variant c.4046G>A (p.Arg1349Gln) in *CACNA1A*, corresponding to p.(Arg1348Gln) in the MANE Select transcript (ClinVar: Variation ID: 380972) [59]. It is classified as pathogenic/likely pathogenic for DEE42, with multiple submitters confirming its deleterious effect [59].

This mutation is a well-characterized gain-of-function alteration affecting the S4 voltage-sensor segment of domain III within the α1A subunit of the CaV2.1 channel [73]. First described by Knierim et al. [74], it removes a highly conserved arginine residue, leading to increased channel open probability, enhanced calcium influx, and neuronal hyperexcitability [74]. It is classically associated with hemiplegic migraine, progressive cerebellar ataxia, and epilepsy [75].

The patient’s phenotype includes focal impaired awareness seizures (onset at 12 years), macrocephaly, craniofacial dysmorphism, café-au-lait spots, cerebellar atrophy, and ataxia-telangiectasia-like features. While cerebellar atrophy and epilepsy are well-established features of *CACNA1A*-related disorders [73,75,76], macrocephaly and craniofacial dysmorphism have been occasionally reported in severe phenotypes [76]. Café-au-lait spots are not typical and may represent an incidental finding or phenotypic expansion that warrants further investigation.

In a cohort of 47 individuals, Lipman et al. [77] reported developmental delay in 96% and epilepsy in 55%, with severe phenotypes linked to gain-of-function mutations, consistent with larger systematic reviews [76]. This variant confirms DEE42 (OMIM: 617106) [39], enabling precise genetic counseling and anticipatory monitoring, though management remains symptomatic, focusing on seizure control and tracking cerebellar decline [73,77].

#### 3.2.6. *GPHN*-Related Spectrum Disorder: The c.1206del, p.(Ala403Hisfs*3) Frameshift Variant

The *GPHN* gene (OMIM: 603930) [39] encodes gephyrin, a scaffolding protein responsible for clustering glycine and GABA type-A receptors at inhibitory synapses. Impairment of GABAergic synaptic inhibition is a well-established pathway in epileptogenesis [78,79].

Patient P04 carries the heterozygous frameshift variant c.1206del (p.Ala403Hisfs3*) in *GPHN*, creating a premature translational stop signal predicted to result in haploinsufficiency of gephyrin. The clinical presentation (generalized convulsions, juvenile myoclonic epilepsy with onset at 9 years, and excessive weight gain) expands the known phenotypic spectrum of *GPHN*-related disorders beyond DEEs and autism spectrum disorder [78,79]. This observation aligns with the concept that heterozygous *GPHN* variants constitute a rare genetic risk factor for common genetic generalized epilepsies [79].

The p.(Ala403Hisfs*3) variant is documented in ClinVar (Variation ID: 2049457) [59] and classified as pathogenic, reinforcing the role of *GPHN* haploinsufficiency in epileptogenesis.

#### 3.2.7. *AFF3*-Related KINSSHIP Syndrome: The c.697G>A, p.(Ala233Thr) Missense Variant

The ALF transcription factor family member AFF3 is a component of the AFF3, a member of the ALF transcription factor family, and is a component of the transcriptional super elongation complex regulating genes involved in neurogenesis and development [39]. De novo missense variants in the degron of *AFF3* (a nine-amino-acid sequence important for its binding to ubiquitin ligase) cause KINSSHIP syndrome [80].

Patient P11 carries the heterozygous missense variant c.697G>A, p.(Ala233Thr) in *AFF3*, a well-characterized pathogenic variant registered in ClinVar (Variation ID: 430951) [59] and shown to occur de novo in multiple individuals with features consistent with KINSSHIP syndrome [80]. The clinical presentation—agenesis of the corpus callosum, seizures, aortic coarctation, patent ductus arteriosus, congenital clubfoot, metatarsal agenesis, polydactyly, facial dysmorphism, and severe developmental delay—aligns with the multisystemic nature of KINSSHIP syndrome [80,81]. The presence of complex congenital anomalies reflects the role of *AFF3* in embryonic development. Approximately 22 cases have been reported worldwide [81], underscoring the rarity of this recently characterized disorder. Molecular confirmation enables accurate genetic counseling and guides multidisciplinary management of the associated congenital anomalies.

#### 3.2.8. *GRIN2A*-Related Focal Epilepsy with Speech Disorder: The Splice-Donor Variant

Pathogenic variants in *GRIN2A*, encoding the GluN2A subunit of the NMDA receptor, are a major cause of focal epilepsy with a speech disorder, with or without intellectual impairment [82,83]. Patient P12 carries a heterozygous splice-donor variant (c.1007+1G>A), predicted to cause loss-of-function (LOF), which has been previously reported in multiple individuals and is classified as pathogenic in ClinVar (Variation ID: 88727) [59].

The clinical presentation (focal epilepsy with onset at 2 years, moderate intellectual disability, behavioral disorders, and fine motor impairment) is consistent with known *GRIN2A*-related disorders [82,83]. EEG findings show isolated and grouped spikes in the temporo-parietal and temporo-median regions with right hemisphere predominance, aligning with centro-temporal discharges typical of this condition [83]. L-serine treatment has shown potential in some loss-of-function cases, although it should be avoided in gain-of-function (GOF) scenarios [82,83].

#### 3.2.9. *CSDE1*-Related Neurodevelopmental Disorder: The c.1087C>T, p(.Arg363*) Nonsense Variant

*CSDE1* encodes an RNA-binding protein critical for neurodevelopment, with disruptive variants linked to autism, intellectual disability, and seizure disorders [39,84]. Patient P13 carries a heterozygous nonsense variant (*CSDE1* c.1087C>T, p.(Arg363*)), leading to a premature stop codon and predicted nonsense-mediated decay (Table 1). This variant is classified as pathogenic in ClinVar (Variation ID: 3901561) [59]. Parental testing was not performed; therefore, its de novo status could not be confirmed. The patient’s presentation, including generalized epilepsy with onset at 1 year, moderate intellectual disability, proportionate dwarfism, microcephaly, and craniofacial dysmorphism, aligns with the emerging *CSDE1*-related spectrum [84]. The findings highlight the impact of *CSDE1* on synaptic function and the utility of comprehensive genetic testing in neurodevelopmental cases.

Additionally, the patient carries a homozygous pathogenic variant in *SPINK5* c.1431-12G>A, a known cause of ichthyosiform erythroderma [39,59], which accounts for the skin manifestations observed in this case. This variant is classified as pathogenic in ClinVar (Variation ID: 372516) [59].

### 3.3. The VUS Cohort: Diagnostic Uncertainty and Future Directions

Variants of uncertain significance (VUSs) represent a substantial challenge in clinical genomics, and our study is no exception, with 13 of 35 tested patients (37.1%) carrying at least one VUS (15 VUSs across 13 patients). This high proportion reflects both the genetic heterogeneity of epilepsy and the limitations of current knowledge regarding the functional impact of many rare missense variants [85]. Importantly, our in silico analyses revealed that 8 out of 15 VUSs had CADD scores exceeding 20, and 8 out of 10 analyzable missense variants had REVEL scores above 0.75, indicating that these variants warrant further investigation to clarify their clinical significance [86,87]. Furthermore, the extremely low or absent allele frequencies in gnomAD (MAF 0.000000 to 0.0000428) support the hypothesis that these are rare, potentially deleterious variants [88].

The identification of de novo variants in four patients (P14, P15, P21, and P22) further supports the need for functional validation [89,90].

Several of these variants are located in genes with strong evidence for epilepsy associations, including *KCNQ2* [91], *CACNA1E* [92], *LGI1* [93], *ARX* [94], and *CNKSR2* [95].

Collectively, our VUS data highlight the need for systematic functional validation, familial segregation studies, and collaborative data sharing to facilitate reclassification [85]. Many of these variants represent strong candidates for future studies using cellular models (e.g., induced pluripotent stem cell-derived neurons) or electrophysiological assays to assess their impact on channel function, synaptic transmission, and neuronal excitability.

### 3.4. Clinical and Phenotypic Differences Between P/LP and VUS Cohorts

The comparative analysis of clinical features between the P/LP and VUS cohorts (Table 5) revealed several noteworthy trends. Intellectual disability was more frequently observed in the P/LP cohort (84.6% vs. 46.2%), suggesting that the presence of a definite pathogenic variant correlates with more profound neurodevelopmental impairment. This is likely attributable to the fact that many P/LP variants are loss-of-function (LOF) mutations in genes critical for brain development and synaptic function, whereas VUS may represent milder or more functionally ambiguous alleles [91,92].

Therapeutic response also differed between the two groups: unsatisfactory seizure control (UR) was observed in 30.8% of VUS patients compared to 15.4% in the P/LP cohort. This counterintuitive finding may be explained by the higher proportion of ion channel variants in the VUS group (*KCNQ2*, *CACNA1A*, *CHRNB2*), which are often associated with inadequate seizure control [96]. In contrast, several P/LP patients with severe genetic etiologies (e.g., Angelman syndrome) achieved good seizure control with appropriate antiseizure medications, highlighting the importance of gene-specific therapeutic tailoring.

Non-neurological and dysmorphic features were both more frequent and more severe in the P/LP cohort, particularly in patients with CNVs or syndromic conditions (KINSSHIP syndrome, 8p inverted duplication/deletion syndrome). Organ malformations (cardiac, skeletal, genitourinary) were exclusively documented in the P/LP group, underscoring the multisystemic nature of pathogenic variants in dosage-sensitive genes [97]. This observation reinforces the clinical principle that the presence of extra-neurological features should prompt a high index of suspicion for a syndromic genetic etiology [85].

### 3.5. Study Limitations

This study has several limitations that should be acknowledged. First, the small sample size (*n* = 13 for the P/LP cohort) limits the generalizability of our findings and precludes formal statistical comparisons. The retrospective design and single-center nature of the study may also introduce selection bias, as patients referred for genetic testing likely represent a more severe or complex subset of the epilepsy population.

Second, the absence of parental samples in most cases hindered segregation analysis, particularly for VUS. The inability to determine whether a variant is inherited or de novo significantly complicates variant interpretation and prevents definitive classification. This issue underscores the importance of including parental testing as a routine component of genetic workups for epilepsy.

Third, the financial burden of genetic testing, which was fully covered by the patients’ families, may have introduced socioeconomic selection bias, potentially excluding patients who could have benefited from molecular diagnosis but lacked the resources to afford testing.

Fourth, while we performed comprehensive in silico analyses, functional validation of a VUS was not conducted due to resource limitations. As a result, many of our VUSs remain provisional and require further experimental characterization to confirm their pathogenicity.

Finally, the lack of longitudinal follow-up data in some patients limits our ability to assess the evolution of clinical phenotypes, treatment responses, and long-term outcomes. Future prospective studies with extended follow-up periods would provide a more robust understanding of genotype–phenotype dynamics.

### 3.6. Comparison with Other Cohorts

The diagnostic yield in our cohort (37.1%) is comparable to large international series, falling within the upper range of previously reported rates (25–40%) in cohorts enriched for early-onset or drug-resistant epilepsies [31,42].

At the national level, two studies have characterized the genetic landscape of pediatric epilepsy in Romania. The first, from the Regional Centre of Medical Genetics Dolj in Craiova, reported a 25% yield in 36 patients with early-onset DEEs using a targeted gene panel [98]. A subsequent study from Timișoara evaluated 140 pediatric patients using gene panels, WES, and whole-genome sequencing, identifying *SCN1A* as the most frequently implicated gene, followed by *SCN2A* and *STXBP1* [99]. Our Moldova-region study, evaluating 35 patients using targeted gene panels and WES, yielded a 37.1% diagnostic rate.

This higher yield, compared to the Dolj and Timișoara cohorts, can be attributed to differences in patient selection. Our inclusion criteria targeted patients with a high suspicion of genetic etiology based on a broader clinical spectrum, including drug-resistant epilepsy, neurodevelopmental comorbidities, and dysmorphic features. In contrast, the Dolj study focused exclusively on early-onset DEEs, whereas the Timișoara study evaluated a more heterogeneous cohort with a lower median age of seizure onset (2.5 years). Furthermore, our combined use of targeted gene panels and WES likely contributed to increased diagnostic sensitivity.

A comparison of the mutational spectrum reveals similarities alongside differences. In the Dolj study, all molecularly confirmed cases involved genes encoding sodium channel subunits: seven with Dravet syndrome (*SCN1A*) and two with genetic epilepsy with febrile seizures plus (*SCN1B*) [98]. Likewise, *SCN1A* was predominant in the Timișoara cohort [99]. Conversely, our cohort displayed broader genetic heterogeneity, with variants in other ion channel genes (*GNAO1*, *KCNB1*, *CACNA1A*), synaptic genes (*GPHN*, *GRIN2A*, *CSDE1*), transcriptional regulators (*AFF3*), and three CNVs. This wider spectrum reflects our more inclusive criteria. Despite these variations, all three national studies consistently underscore *SCN1A* as the predominant gene driving monogenic epilepsies across Romania.

Finally, the identification of a novel pathogenic variant (*SCN1A* c.2870G>A, p.(Trp957*)) may reflect population-specific allelic diversity that warrants further investigation in larger cohorts, emphasizing that studying diverse populations is essential to mitigate diagnostic disparities. Moreover, the observed clinical heterogeneity among patients sharing identical genetic diagnoses supports the hypothesis that common genetic modifiers influence disease expression, suggesting that future investigations should integrate rare variant analyses with polygenic risk scores [100].

### 3.7. Future Directions and Clinical Implications

Future directions should include prospective studies with larger cohorts, expanded access to whole-genome sequencing (WGS) for negative cases, systematic functional validation of VUSs [85], and integration of polygenic risk scores (PRSs) to refine risk stratification and phenotypic prediction [100]. Recent preclinical advances in neuron-specific gene replacement have demonstrated dose-dependent and durable rescue of disease phenotypes in models of genetic epileptic encephalopathies [101]. Activity-dependent gene therapy represents a particularly promising strategy for genetically defined epilepsies, as it offers improved temporal and spatial specificity by targeting only the hyperactive neurons that drive pathological network activity while sparing surrounding healthy circuits. By using activity-dependent promoters such as c-fos to drive the expression of inhibitory effectors (e.g., the *KCNA1* potassium channel), this approach has demonstrated sustained suppression of spontaneous seizures—around an 80% reduction in preclinical models—without impairing normal cognition [102]. Beyond genetic testing, emerging biomarkers may complement genomic findings in difficult-to-classify or treatment-resistant cases. Fast-ripple activity represents an electrophysiological marker of pathological network excitability that may aid in identifying the epileptogenic zone, while glutamate receptor–reactive antibodies have been reported in enigmatic intractable epilepsy as potential immune biomarkers that could guide immunotherapy in selected patients [103,104]. Collaborative efforts across centers and countries will be essential to build sufficiently powered datasets and to translate genetic discoveries into improved clinical outcomes for patients with epilepsy.

## 4. Materials and Methods

### 4.1. Study Design and Patient Selection

This retrospective study was conducted at the Neurology Clinic and the Regional Genetics Center of St. Mary Emergency Children Hospital, Iași, Romania, serving patients from the Moldova region of Romania. To our knowledge, this is the first molecular study to characterize the genetic landscape of epilepsy in pediatric patients from this region. The study included patients diagnosed with epilepsy between January 2024 and June 2026. A total of 159 patients with epilepsy were initially screened. Of these, 124 were excluded due to epilepsy secondary to brain malformations (*n* = 19), acquired structural lesions (e.g., post-traumatic or ischemic) (*n* = 62), metabolic disorders (*n* = 5), or other identifiable acquired etiologies (*n* = 38), including central nervous system infections, brain tumors, and autoimmune encephalitis.

The remaining 35 patients, in whom a primary genetic etiology was strongly suspected based on clinical presentation, were referred for extensive molecular testing. Patient selection for genetic testing was based on the recommendations of the ILAE Genetics Commission [42] and the National Society of Genetic Counselors (NSGC) practice guideline for unexplained epilepsies [34]. Patients were eligible if they met at least one of the following criteria: (1) seizure onset within the first two years of life; (2) drug-resistant epilepsy, defined as failure of at least two appropriate antiseizure medications; (3) presence of intellectual disability, autism spectrum disorder, or global developmental delay; (4) dysmorphic features or congenital anomalies suggestive of a genetic syndrome; or (5) a positive family history of epilepsy or neurodevelopmental disorders in first-degree relatives.

Genetic analyses were performed using either a targeted epilepsy gene panel or whole-exome sequencing (WES) at Blueprint Genetics (Espoo, Finland) or Invitae (San Francisco, CA, USA). Targeted epilepsy gene panels were used for patients with typical electroclinical phenotypes, while WES was reserved for atypical, complex cases or those with systemic manifestations. It is important to note that the costs of these analyses were fully covered by the patients’ families, as extended genetic testing for epilepsy is not reimbursed by the Romanian national health system.

### 4.2. Molecular Genetic Testing and Variant Classification

Genetic testing was performed using next-generation sequencing (NGS) with Illumina technology (San Diego, CA, USA) at the aforementioned commercial laboratories. Total genomic DNA was extracted from peripheral blood samples using a bead-based method, and DNA quantity was assessed fluorometrically. Qualified genomic DNA samples were randomly fragmented using non-contact, isothermal sonochemistry, followed by ligation of sequencing adapters to both ends of the DNA fragments to prepare a sequencing library. Size selection was performed using a bead-based method to ensure optimal template size, and the library was amplified by polymerase chain reaction (PCR). Regions of interest (exons and intronic targets) were captured using a hybridization-based target capture method, and library quality was assessed to ensure correct template size and quantity. Ready libraries were sequenced using Illumina’s sequencing-by-synthesis method with paired-end reads (2 × 150 bases). The average target coverage was >100×, with >95% of the target bases covered at a minimum depth of 20×.

Primary data analysis was performed using Illumina’s proprietary software v2.20 (bcl2fastq), and raw sequencing data were converted to FASTQ format. Sequence reads were mapped to the human reference genome (GRCh37/hg19) using the Burrows–Wheeler Aligner (BWA MEM). Variant annotation was performed using VcfAnno and VEP, with reference to public variant databases, including gnomAD, ClinVar, and HGMD. For missense variants, in silico prediction tools, such as SIFT, PolyPhen, MutationTaster, CADD, and REVEL, were used to assist with variant classification. All clinically relevant variants (including P/LP variants and selected VUSs) were confirmed by Sanger sequencing.

Copy number variations (CNVs), defined as single-exon or larger deletions or duplications, were detected using the proprietary bioinformatics pipelines of the commercial laboratories (Blueprint Genetics, Espoo, Finland; Invitae, San Francisco, CA, USA), which analyze read-depth coverage across target regions and compare it to a reference cohort of samples processed on the same platform. All reported CNVs were confirmed by an orthogonal method according to each laboratory’s standard protocol. Specifically, CNVs covering fewer than 10 exons were confirmed using digital PCR (dPCR), as explicitly stated in the clinical reports. Larger CNVs, including the 15q11.2-q13.1 deletion and the 17q21.31 microdeletion, were confirmed by the reporting laboratory using their validated internal protocols. It is important to note that NGS-based CNV analysis has inherent limitations: it cannot determine exact breakpoints, does not detect balanced translocations or complex inversions, and may not reliably detect low-level mosaicism. Regarding the 8p rearrangement in patient P09, the deletion and duplication were detected by WES, and the diagnosis of inverted duplication/deletion of 8p was established based on the characteristic pattern of terminal deletion and inverted duplication, consistent with published literature [71,72]. Direct confirmation of the inversion component by FISH or array CGH was not available. However, the clinical phenotype of the patient, including severe developmental delay, corpus callosum anomalies, complex cardiac malformation, and seizures, is highly consistent with this diagnosis [72], providing further support for the molecular findings.

For the 15q11.2-q13.1 deletion identified in patient P07, methylation analysis was performed to establish the parent of origin, confirming the maternal origin of the deletion, consistent with a diagnosis of Angelman syndrome. We evaluated the pathogenicity of all detected variants in accordance with the American College of Medical Genetics and Genomics and the Association for Molecular Pathology (ACMG/AMP) recommendations [85], while also reviewing entries in HGMD Professional [105] and ClinVar [59]. The resulting evidence enabled us to designate each variant as belonging to one of five groups: pathogenic, likely pathogenic, variant of uncertain significance (VUS), likely benign, or benign. The specific ACMG/AMP evidence codes used for each P/LP variant are provided in Table S2 (Supplementary Material).

### 4.3. Clinical Phenotype Assessment and Descriptive Comparative Analysis

Clinical data were collected retrospectively from medical records. For each patient, we documented demographic information, clinical manifestations, age at seizure onset, seizure types, electroencephalography (EEG) findings, brain magnetic resonance imaging (MRI) results, treatment response, presence of intellectual disability, other systemic manifestations, and family history. Intellectual disability was assessed clinically and categorized as mild (IQ 50–70), moderate (IQ 35–49), or severe (IQ < 35) based on medical records and standardized developmental assessments when available (e.g., WISC, WPPSI), in accordance with the American Psychiatric Association (APA) criteria [106].

Treatment response was classified as good (GR: seizure freedom or ≥50% reduction in seizure frequency), partial (PR: 25–49% reduction), or unsatisfactory (UR: <25% reduction, no change, or worsening). This classification was adapted from previously established seizure outcome schemes that utilize graduated response thresholds, including the six-class categorization (0: <25%, 1: 25–50%, 2: 50–75%, 3: 75–90%, 4: ≥90%, 5: seizure-free) proposed by Järvenpää et al. [107]. The ≥50% threshold is consistent with standard definitions of clinical response used in epilepsy trials [108,109]. It is important to note that these categories (GR, PR, UR) describe percentage seizure reduction and are not equivalent to the formal ILAE definition of drug-resistant epilepsy. Genetic counseling was provided to all families, and family trees (pedigrees) were constructed for each patient.

Given the exploratory nature of this study and the limited sample size (*n* = 13 for the P/LP cohort), no formal statistical hypothesis testing was performed. Instead, descriptive statistics (frequencies, percentages, means, and ranges) were used to summarize the clinical characteristics of the cohort. A comparative descriptive analysis was conducted between the group of patients carrying P/LP variants and the group harboring VUSs, focusing on the overlap and divergence in their respective clinical presentations. The results were interpreted in the context of published data, with emphasis on each patient’s distinctive features within their family setting and on potential genotype–phenotype correlations that require further exploration in larger cohorts.

### 4.4. Ethics and Informed Consent

This study was conducted in accordance with the principles of the Declaration of Helsinki and was approved by the Ethics Committee of St. Mary Emergency Children Hospital, Iași, Romania (Certificate no. 28726/01.09.2026). Written informed consent was obtained from all subjects involved in the study (or from their parents/legal guardians for minors).

## 5. Conclusions

This study provides a detailed characterization of a Romanian cohort of patients with suspected genetic epilepsy, demonstrating a diagnostic yield of 37.1% through targeted gene panels or whole-exome sequencing (WES). Our findings reaffirm that pathogenic variants in ion channel genes, particularly *SCN1A*, *KCNB1*, and *GNAO1*, are the predominant causes of early-onset, drug-resistant epileptic encephalopathies, while copy number variants (CNVs) contribute to a subset of syndromic cases with prominent extra-neurological manifestations. Notably, the identification of a novel pathogenic variant (*SCN1A* c.2870G>A, p.(Trp957*)) expands the mutational spectrum of epilepsy-associated genes and may reflect population-specific allelic diversity that warrants further investigation in larger cohorts. Furthermore, the characterization of 15 variants of uncertain significance (VUS) across 13 patients, many presenting high in silico support and de novo occurrences, offers a valuable resource for future reclassification efforts.

This emphasizes the urgent need for functional validation, familial segregation analyses, and collaborative data sharing. While our results underscore the clinical utility of genetic testing in guiding therapeutic decisions, enabling accurate genetic counseling, and improving prognostic accuracy, the substantial proportion of VUSs identified within the cohort reflects ongoing challenges in variant interpretation and the continuous need to refine classification criteria. As the first molecular data on genetic epilepsies in a pediatric cohort from the Moldova region of Romania and the third nationwide following reports from Dolj (Craiova) [98] and Timișoara [99], this study provides essential foundational insights. Given the exploratory nature of these findings and the relatively small number of molecularly confirmed cases (*n* = 13), larger, multicenter cohort studies are essential to establish a comprehensive mutational profile of genetic epilepsies in the Romanian population and to validate the genotype–phenotype findings reported here.

Key messages for clinical practice: 1. Extended genetic testing should be considered an early diagnostic option in children with early-onset, unexplained, or drug-resistant epilepsy. 2. The presence of dysmorphic features, growth abnormalities, or congenital anomalies should prompt CNV analysis, as these often indicate syndromic etiologies. 3. Variants of uncertain significance (VUSs) should not be disregarded; periodic re-evaluation as new evidence emerges is essential, and de novo status or high in silico scores should prioritize variants for functional studies. 4. The heavy financial burden of genetic testing, fully covered by patients’ families in this cohort, highlights an urgent systemic need to address cost barriers, ensuring equitable access to precision diagnostics and enabling the integration of molecular testing into standard pediatric neurological care.

## Figures and Tables

**Table 1:** Genotype–phenotype findings in epilepsy: a study of a Romanian patient cohort.

Patient ID	Gene	Transcript	Variant (HGVS)	Zygosity	Testing Method	Variant Previously Reported	Effect/Pathogenicity (ACMG)	Inheritance	Family History of Epilepsy	Diagnosis (OMIM) [39]
P01 (M.I.)	*KCNB1*	NM_004975.4	c.1107G>A p.(Trp369*)	Hz	Panel	Known	Stop_gained/ Pathogenic	AD	Positive	DEE26 (OMIM: 616056)
P02 (M.P.)	*GNAO1*	NM_020988.2	c.607G>A (p.Gly203Arg)	Hz	Panel	Known	Missense /Pathogenic	AD	Negative	DEE17 (OMIM: 615473)
P03 (C.I.)	*GNAO1*	NM_020988.3	c.143C>T p.(Thr48Ile)	Hz	WES	Known	Missense /Pathogenic	AD	Negative	DEE17 (OMIM: 615473)
P04 (B.T.)	*GPHN*	NM_020806.4	c.1206del p.(Ala403Hisfs*3)	Hz	Panel	Known	Frameshift/ Pathogenic	AD	Negative	*GPHN*-related spectrum disorder (including DEE) (PMID: 23393157) (OMIM: 603930)
P05 (P.C.)	*SCN1A*	NM_001165963.4	c.1837C>T p.(Arg613Ter) or R613X	Hz	Panel	Known	Nonsense/Pathogenic	AD	Negative	DEE6 (Dravet syndrome) (OMIM: 607208)
P06 (B.A)	*SCN1A*	NM_001165963.1	c.2870G>A p.(Trp957*)	Hz	Panel	New	Nonsense/ Pathogenic	AD	Negative	DEE6 (Dravet syndrome) (OMIM: 607208)
P07 (B.D.)	-	-	del(15)(q11.2q13.1), including (GABRB3 and UBE3A)	Hz	Panel	Known	Deletion/Pathogenic	Imprinting (Maternal)	Negative	AS (OMIM: 105830)
P08 (N.V.)	*KANSL1*	-	del(17)(q21.31) (exons 3–15)	Hz	WES	Known	Deletion/ Pathogenic	AD	Negative	KVS (OMIM: 610443)
P09 (G.I.)	-	-	seq[GRCh37] del(8)(p23.3p23.1), chr8:g.116025_6794488del; seq[GRCh37] dup(8)(p23.1p11.21), chr8:g.12583147_41836370dup	-	WES	Known	Deletion/ Pathogenic Duplication/Pathogenic	Chromosomal	Negative	8p inverted duplication/deletion syndrome
P10 (C.M.)	*CACNA1A*	NM_023035.3	c.4046G>A p.(Arg1349Gln)	Hz	WES	Known	Missense/ Pathogenic	AD	Negative	DEE42 (OMIM: 617106)
P11 (A.M)	*AFF3*	NM_001386135.1	c.697G>A p.(Ala233Thr)	Hz	WES	Known	Missense/ Pathogenic	AD	Negative	KINS (OMIM: 619297)
P12 (B.M.)	*GRIN2A*	NM_000833.3:	c.1007+1G>A p.(?)	Hz	WES	Known	Splice donor/Pathogenic	AD	Negative	FESD (OMIM: 245570)
P13 (T.L.)	*CSDE1*	NM_001130523.3	c.1087C>T p.(Arg363*)	Hz	WES	Known	Stop_gained/ Likely pathogenic	AD	Negative	CSDE1-related complex neurodevelopmental disorder (OMIM: 191510)

Hz: heterozygous genotype; Ho: homozygous genotype; AD: autosomal dominant; DEE: developmental and epileptic encephalopathy; AS: Angelman syndrome (OMIM: 105830); KVS: Koolen–de Vries syndrome/17q21.31 microdeletion syndrome (OMIM: 610443); KINS: KINSSHIP syndrome (OMIM: 619297); FESD: epilepsy, focal, with speech disorder and with or without impaired intellectual development (OMIM: 245570); P07: seq[GRCh37] del(15)(q11.2q13.1), chr15:g.23006160_28566663del (including *GABRB3* and *UBE3A*); P08: Seq[GRCh37] del(17)(q21.31), chr17:g.43861645_44172184del (exons 3–15 of *KANSL1*); P09: seq[GRCh37] del(8)(p23.3p23.1), chr8:g.116025_6794488del; seq[GRCh37] dup(8)(p23.1p11.21), chr8:g.12583147_41836370dup; WES: whole-exome sequencing.

**Table 2:** Variants of uncertain significance (VUSs) identified in an epilepsy cohort—in silico predictions and phenotypic associations.

Patient ID	Gene	Transcript	Variant (HGVS)	Genotype	Testing Method	gnomAD Max MAF	CADD	REVEL	SIFT	Variant Previously Reported	Phenotype
P14 (S.V.)	*KCNQ2*	NM_172107.4	c.317G>A p.(Cys106Tyr)	Hz	Panel	0.000001	28.3	0.92	Deleterious	New	BFNS1/ DEE7
P15 (H.S.)	*LGI1*	NM_005097.4	c.1597C>T p.(Arg533Cys)	Hz	Panel	0.000001	26.1	0.81	Deleterious	Known	AD-ETL1
P16 (D.M.)	*CNKSR2*	NM_001330653.2	c.1709G>A p.(Arg570His)	Hmz	WES	0.000000	25.9	0.90	Deleterious	New	MRXSHG
P17 (N.B.)	*CACNA1E*	NM_000721.4	c.3275C>T p.(Thr1092Met)	Hz	Panel	0.000004	25.7	0.78	Deleterious	Known	DEE69
P18 (P.V.)	*TBC1D24*	NM_001199107.2	c.448G>A p.(Glu150Lys)	Ho	Panel	0.000008	24.5	0.85	Deleterious	Known	FIME
P19 (S.L.)	*TBCD*	NM_005993.5	c.3250G>T p.(Asp1084Tyr)	Ho	Panel	0.000000	24.3	0.88	Deleterious	New	PEBAT
P20 (P.S.)	*HECW2*	NM_020760.4	c.4492C>A p.(Gln1498Lys)	Hz	Panel	0.000002	22.1	NA	Tolerated	New	NDHSAL
P21 (T.R.)	*RORA*	NM_134261.3	c.615C>G p.(Asp205Glu)	Hz	WES	0.000000	20.4	0.62	Tolerated	New	IDDECA
P22 (P.R.)	*ANKRD11*	NM_013275.6	c.6209A>G p.(Lys2070Arg)	Hz	WES	0.000003	18.2	0.45	Tolerated	New	KBGS
P23 (C.S.)	*TRPM3*	NM_020952.6	c.1262A>G p.(Tyr421Cys)	Hz	WES	0.0000328	NA	NA	NA	New	NEDFSS
P23 (C.S.)	*POLR2A*	NM_000937.5	c.4720T>C p.(Ser1574Pro)	Hz	WES	NA	NA	NA	NA	New	NEDHIB
P24 (D.D.)	*ARX*	NM_139058.3	c.499G>A p.(Val167Met)	Hmz	WES	0.0000428	NA	NA	NA	New	DEE1
P25 (L.S.)	*CACNA1A*	NM_001127221.1	c.1954G>A p.(Asp652Asn)	Hz	WES	NA	NA	NA	NA	Known	DEE42
P26 (B.E.)	*CHRNB2*	NM_000748.3	c.1082T>C p.(Leu361Pro)	Hz	Panel	NA	NA	0.94	NA	Known	ENFL3
P26 (B.E.)	*CNKSR2*	NM_014927.3	c.1154A>T p.(His385Leu)	Hz	Panel	NA	NA	0.870	NA	New	MRXSHG

VUS: variant of uncertain significance; Hz: heterozygous; Ho: homozygous; Hmz: Hemizygous (X-linked); MAF: maximum allele frequency; gnomAD: Genome Aggregation Database; CADD: Combined Annotation Dependent Depletion; REVEL: Rare Exome Variant Ensemble Learner; SIFT: Sorting Intolerant From Tolerant; BFNS1: benign familial neonatal seizures-1 (OMIM: 121200); DEE7: developmental and epileptic encephalopathy-7 (OMIM: 613720); AD-ETL1: autosomal dominant familial temporal lobe epilepsy-1 (OMIM: 600512); MRXSHG: Houge type of X-linked syndromic intellectual developmental disorder (OMIM: 301008); DEE69: developmental and epileptic encephalopathy-69 (OMIM: 618285); FIME: familial infantile myoclonic epilepsy (OMIM: 605021); PEBAT: early-onset progressive encephalopathy with brain atrophy and thin corpus callosum (OMIM: 617193); NA: not available; NDHSAL: neurodevelopmental disorder with hypotonia, seizures, and absent language (OMIM: 617268); IDDECA: intellectual developmental disorder with or without epilepsy or cerebellar ataxia (OMIM: 618060); KBGS: KBG syndrome (OMIM: 148050); NEDFSS: neurodevelopmental disorder with hypotonia, dysmorphic facies, and skeletal anomalies, with or without seizures (OMIM: 620224); NEDHIB: neurodevelopmental disorder with hypotonia and variable intellectual and behavioral abnormalities (OMIM: 618603); DEE1: developmental and epileptic encephalopathy-1 (OMIM: 308350); DEE42: developmental and epileptic encephalopathy-42 (OMIM: 617106); ENFL3: epilepsy, nocturnal frontal lobe, type 3 (OMIM: 605375); HLD1: Pelizaeus–Merzbacher disease (X-linked recessive hypomyelinating leukodystrophy) (OMIM: 312080); WES: whole-exome sequencing.

**Table 3:** Clinical features of molecularly confirmed epilepsy patients (P01–P13).

Patient ID	Age at Seizure Onset	Seizure Type (s)	EEG Findings *	Treatment Response	Intellectual Disability	Other Manifestations	Diagnosis (Gene)
P01 (M.I.)	1y7mo	FPC	Spike–slow wave complex, left F-O	UR	SID	Autism, short stature (−2.5 SD), low weight (−2.98 SD), frontal bossing, craniofacial dysmorphism	DEE26 (*KCNB1)*
P02 (M.P.)	3mo	FPC	Spike left C-T	GR	MID	Severe motor delay, nystagmus, microcephaly, PDA, hypotrophy	DEE17 (*GNAO1*)
P03 (C.I.)	2mo	ES	Hypsarrhythmia	GR	SID	Proportionate dwarfism (−3 SD), microcephaly (−3.58 SD), GER, Sandifer syndrome, dystonic postures, severe motor delay	WS/DEE17 (*GNAO1*)
P04 (B.T.)	10y	GTGS, GMS	Generalized spike–slow wave complex	PR	-	Weight hypertrophy	JME/*GPHN*-related spectrum disorder
P05 (P.C.)	1y	FS, GTCS, FPC	Generalized spike–slow wave complex	PR	MID	Autism, language delay	DEE6/Dravet syndrome (*SCN1A*)
P06 (B.A.)	11mo	FS, FPC, GTCS, GM	Generalized spike–slow wave complex	PR	MID	Autism, language delay	DEE6/Dravet syndrome (*SCN1A*)
P07 (B.D.)	2y3mo	GTCS	Generalized slow wave	GR	MID	Motor and language delay, ataxic movements	Angelman syndrome (15q11.2-q13.1 deletion)
P08 (N.V.)	9mo	GTCS	Generalized spike–slow wave complex	UR	SID	Craniofacial dysmorphia, cryptorchidism, ASD, hypotonia, joint/skin laxity, motor delay	Koolen–de Vries syndrome (17q21.31 deletion)
P09 (G.I.)	1y	GTCS	Generalized spike–slow wave complex	PR	SID	Proportionate dwarfism, craniofacial dysmorphia (ptosis, dental malposition), spastic paraparesis, extrapyramidal syndrome, ataxia, partial ACC, complex cardiac malformation	8p inverted duplication/deletion syndrome
P10 (C.M.)	12y	FIC	Generalized spike–slow wave complex	GR	-	Macrocephaly, craniofacial dysmorphism, café-au-lait spots, multiple nevi, cerebellar atrophy	DEE42 (*CACNA1A*)
P11 (A.M.)	5mo	FPC, FBTC	Right T-O spikes	GR	SID	Language delay, Aortic coarctation, PDA, inguinal hernia, congenital clubfoot, metatarsal agenesis (left foot), polydactyly (left foot), simian crease (left hand), facial dysmorphism, motor delay	KINS syndrome (*AFF3*)
P12 (B.M.)	2y	FPC	Isolated/grouped spikes, T-P, T-M, C-region, right hemisphere predominance	PR	MID	DIL secondary to ASM, BD, FMI	FESD (*GRIN2A*)
P13 (T.L.)	1y	GTCS	Generalized spike–slow wave complex	GR	MID	Proportionate dwarfism, microcephaly, craniofacial dysmorphism, congenital ichthyosiform erythroderma	*CSDE1*-related complex neurodevelopmental disorder + Netherton syndrome (*SPINK5*-related)

* Seizure types presented are those documented in the patients’ clinical records and may differ from the most commonly reported phenotypes for each respective gene; FIC: focal impairment consciousness seizure; FPC: focal preserved consciousness seizure; FS: febrile seizures; ES: epileptic spasms; GM: generalized myoclonic seizures; GTCS: generalized tonic–clonic seizures; MS: myoclonic seizures; FBTC: focal-to-bilateral tonic–clonic seizure; SE: infantile spasms (spasms epileptic); FE: focal epilepsy; F-O: fronto-occipital; C-T: centro-temporal; T-O: tempo-occipital; T-P: temporo-parietal; T-M: temporo-median; C: central. Treatment response: UR: unsatisfactory response; GR: good response; PR: partial response; MID: mild intellectual disability; SID: severe intellectual disability; BD: behavioral disorders; FMI: fine motor impairment; WS: West syndrome; JME: juvenile myoclonic epilepsy; FESD: focal epilepsy with speech disorder and with or without impaired intellectual development; DEE: developmental and epileptic encephalopathy; ASD: atrial septal defect; PDA: patent ductus arteriosus; ACC: agenesis of corpus callosum; GER: gastroesophageal reflux; DIL: drug-induced liver injury; ASM: antiseizure medication; SD: standard deviation.; y: years; mo: months.

**Table 4:** Clinical features of epilepsy patients with variants of uncertain significance (VUSs).

Patient ID	Age at Seizure Onset	Seizure Type (s)	EEG Findings *	Treatment Response	Intellectual Disability	Other Manifestations	Diagnosis (Gene)
P14 (S.V.)	2y5mo	FPC	Generalized spike–slow wave complex	UR	MID	Language delay	BFNS1/DEE7 (*KCNQ2*)
P15 (H.S.)	6y	AA	Generalized spike–slow wave complex 3c/sec, polyspike–slow wave	UR	-	-	AD-ETL1 (*LGI1*)
P16 (D.M.)	1y10mo	FS, GTCS, AA	Biphasic spikes right C-T	PR	MID	Autism, ataxic syndrome, language delay	MRXSHG (*CNKSR2*)
P17 (N.B.)	3mo	FPC	Right T-O spikes	GR	-	Language delay	DEE69 (*CACNA1E*)
P18 (P.V.)	4y8mo	EAM	Slow waves C-T bilateral	GR	-	-	FIME (*TBC1D24)*
P19 (S.L.)	2mo	GTCS	Bilateral irritative background T bilateral	GR	-	-	PEBAT (*TBCD*)
P20 (P.S.)	6mo	FPC	Spike left hemisphere	PR	MID	Motor delay	NDHSAL (*HECW2*)
P21 (T.R.)	3y	AA GTCS	Spike–slow wave complex	GR	MID	Autism, language delay, ADHD, craniofacial dysmorphism	IDDECA (*RORA*)
P22 (P.R.)	5y	FPC	Spike–slow wave complex, bilateral spikes	GR	SID	Motor delay	KBGS (*ANKRD11*)
P23 (C.S.)	10mo	FS, GTCS, GT, AA	Biphasic spikes right C-T	PR	-	Motor delay, Language delay	NEDFSS/NEDHIB (*TRPM3*/*POLR2A*)
P24 (D.D.)	2y	FPC	Rhythmic sharp wave discharges, F-T region	GR	MID	Autism, language delay, ADHD, craniofacial dysmorphism, joint laxity	DEE1 (ARX)
P25 (L.S.)	13y	AA	Generalized, bilateral, synchronous spike–wave complexes	UR	-	Juvenile arthritis, gastritis, GERD	DEE42 (CACNA1A)
P26 (B.E.)	8mo	GM	Left frontal sharp wave during wakefulness, multiple spike-and-wave	UR	-	Weight hypertrophy (+2.9 SD), craniofacial dysmorphism	ENFL3 (*CHRNB2*)/MRXSHG (*CNKSR2*)

* Seizure types presented are those documented in the patients’ clinical records and may differ from the most commonly reported phenotypes for each respective gene; FPC: focal preserved consciousness seizure; FS: febrile seizures; GTCS: generalized tonic–clonic seizures; GA: generalized absence; AA: atypical absence seizures; EAM: eyelid myoclonia with/without absence; GT: generalized tonic seizure; GM: generalized myoclonic seizures; C-T: centro-temporal; T-O: tempo-occipital; F-T: fronto-temporal; T: temporal; UR: unsatisfactory response; GR: good response; PR: partial response; MID: mild intellectual disability; SID: severe intellectual disability; ADHD: attention deficit hyperactivity disorder; GERD: gastroesophageal reflux disease; SD: standard deviation; BFNS1: benign familial neonatal seizures-1 (OMIM: 121200); DEE7: developmental and epileptic encephalopathy-7 (OMIM: 613720); AD-ETL1: autosomal dominant familial temporal lobe epilepsy-1 (OMIM: 600512); MRXSHG: Houge type of X-linked syndromic intellectual developmental disorder (OMIM: 301008); DEE69: developmental and epileptic encephalopathy-69 (OMIM: 618285); FIME: familial infantile myoclonic epilepsy (OMIM: 605021); PEBAT: early-onset progressive encephalopathy with brain atrophy and thin corpus callosum (OMIM: 617193); NDHSAL: neurodevelopmental disorder with hypotonia, seizures, and absent language (OMIM: 617268); IDDECA: intellectual developmental disorder with or without epilepsy or cerebellar ataxia (OMIM: 618060); KBGS: KBG syndrome (OMIM: 148050); NEDFSS: neurodevelopmental disorder with hypotonia, dysmorphic facies, and skeletal anomalies, with or without seizures (OMIM: 620224); NEDHIB: neurodevelopmental disorder with hypotonia and variable intellectual and behavioral abnormalities (OMIM: 618603); DEE1: developmental and epileptic encephalopathy-1 (OMIM: 308350); DEE42: developmental and epileptic encephalopathy-42 (OMIM: 617106); ENFL3: epilepsy, nocturnal frontal lobe, type 3 (OMIM: 605375).

**Table 5:** Comparative clinical features of the molecularly confirmed (P/LP) and VUS epilepsy cohorts.

Clinical Parameter	P/LP Cohort (Molecularly Confirmed) (*n* = 13)	VUS Cohort (Uncertain Significance) (*n* = 13)
Age at onset (range)	Early infancy (2 months)–Adolescence (12 years)	Neonatal/Infancy (2 months)–Adolescence (13 years)
Predominant seizure types	FPC, GTCS, Infantile spasms (West syndrome)	FPC, GTCS, atypical absences, myoclonic seizures
Good Response (GR)	46.2% (*n* = 6/13)	46.2% (*n* = 6/13)
Partial Response (PR)	30.8% (*n* = 4/13)	23.1% (*n* = 3/13)
Unsatisfactory Treatment Response (UR)	15.4% (*n* = 2/13)–P01 (*KCNB1*), P08 (*KANSL1*)	30.8% (*n* = 4/13)–P14 (*KCNQ2*), P15 (LGI1), P25 (*CACNA1A*), P26 (*CHRNB2*)
Intellectual Disability (ID)	84.6% (*n* = 11/13) (MID/SID)	46.2% (*n* = 6/13) (MID/SID) *
Neuropsychological Comorbidities	Autism, Severe language delay	Autism, ADHD, language delay

* In the VUS cohort, 6 patients presented with intellectual disability (MID or SID), while an additional 2 patients had language delay without formal ID; MID: mild intellectual disability; SID: severe intellectual disability; VUS: variant of uncertain significance; treatment response is defined as follows: GR (≥50% reduction or seizure freedom); PR (25–49% reduction); UR (<25% reduction or no change).

## Data Availability

The original contributions presented in this study are included in the article. Further inquiries can be directed to the corresponding author.

## Supplementary Materials

The following supporting information can be downloaded at: https://www.mdpi.com/article/10.3390/ijms27198878/s1.

## Abbreviations

The following abbreviations are used in this manuscript:

ACMG	American College of Medical Genetics and Genomics
AD	Autosomal dominant
CNV	Copy number variation
DEE	Developmental and epileptic encephalopathy
FESD	Focal epilepsy with speech disorder
GGE	Genetic generalized epilepsy
gnomAD	Genome Aggregation Database
GOF	Gain-of-function
GWAS	Genome-wide association study
HGMD	Human Gene Mutation Database
HGVS	Human Genome Variation Society
ILAE	International League Against Epilepsy
LOF	Loss-of-function
MAF	Maximum allele frequency
MLPA	Multiplex ligation-dependent probe amplification
NGS	Next-generation sequencing
P/LP	Pathogenic/likely pathogenic
PCR	Polymerase chain reaction
PRS	Polygenic risk score
SNV	Single nucleotide variant
VUS	Variant of uncertain significance
WES	Whole-exome sequencing
WGS	Whole-genome sequencing
