# Supplementary material for: Precision Medicine in Epilepsy: Diagnostic Yield by Next-Generation Sequencing and Genotype–Phenotype Findings in a Romanian Cohort
Source: Int J Mol Sci. 2026 Oct 6;27(19):8878. doi: 10.3390/ijms27198878 (PMC13665156; doi:10.3390/ijms27198878)
Supplement: Supplementary file 1 [file ijms-27-08878-s001.zip › ijms-4571707-supplementary.pdf]

Table S1. Distribution of P/LP and VUS by testing method.

Testing method	Total tested	P/LP	VUS	Negative	P/LP yield	Total reportable yield
WES	17	7 (41.2%)	6 (35.3%)	4 (23.5%)	41.2%	76.5%
Targeted panel	18	6 (33.3%)	7 (38.9%)	5 (27.8%)	33.3%	72.2%
Total	35	13 (37.1%)	13 (37.1%)	9 (25.7%)	37.1%	74.3%

Table S2. ACMG/AMP Evidence Codes for P/LP Variants.

Patient ID	Gene	Variant	Classification	ACMG/AMP Codes
P01	*KCNB1*	c.1107G>A p.(Trp369*)	Pathogenic	PVS1, PM2, PP3, PP5
P02	*GNAO1*	c.607G>A p.(Gly203Arg)	Pathogenic	PS1, PM2, PP3, PP5, PS3
P03	*GNAO1*	c.143C>T p.(Thr48Ile)	Pathogenic	PS1, PM2, PP3, PP5, PS3
P04	*GPHN*	c.1206del p.(Ala403Hisfs*3)	Pathogenic	PVS1, PM2, PP5
P05	*SCN1A*	c.1837C>T p.(Arg613Ter)	Pathogenic	PVS1, PS1, PM2, PP5
P06	*SCN1A*	c.2870G>A p.(Trp957*)	Pathogenic	PVS1, PM2, PP3
P07	15q11.2-q13.1 del	del(15)(q11.2q13.1)	Pathogenic	PVS1, PS1, PM2, PP5
P08	*KANSL1*	17q21.31 microdeletion	Pathogenic	PVS1, PS1, PM2, PP5
P09	8p inv dup/del	del(8)(p23.3p23.1), dup(8)(p23.1p11.21)	Pathogenic	PVS1, PS1, PM2, PP5
P10	*CACNA1A*	c.4046G>A p.(Arg1349Gln)	Pathogenic	PS1, PM2, PM5, PP3, PP5
P11	*AFF3*	c.697G>A p.(Ala233Thr)	Pathogenic	PS1, PM2, PP3, PP5
P12	*GRIN2A*	c.1007+1G>A p.(?)	Pathogenic	PVS1, PS1, PM2, PP5
P13	*CSDE1*	c.1087C>T p.(Arg363*)	Likely pathogenic	PVS1, PM2, PP5

PVS1: Pathogenic Very Strong 1 (null variant in a gene where loss-of-function is a known disease mechanism); PS1: Pathogenic Strong 1 (same amino acid change as a known pathogenic variant); PS3: Pathogenic Strong 3 (well-established functional studies confirm a deleterious effect); PM2: Pathogenic Moderate 2 (absent from gnomAD or extremely low frequency); PM5: Pathogenic Moderate 5 (different amino acid change at the same codon as a known pathogenic variant); PP3: Pathogenic Supporting 3 (multiple in silico tools predict a deleterious effect); PP5: Pathogenic Supporting 5 (variant reported as pathogenic in a reputable database, such as ClinVar or HGMD);The novel *SCN1A c.2870G>A, p.(Trp957*) variant was classified as pathogenic based on PVS1 (nonsense variant), PM2 (absent from gnomAD), and PP3 (multiple in silico tools predict a deleterious effect).
